# A Hard Pill to Swallow: Dysphagia-Associated Factors Following Adult Cervical Deformity Surgery

**DOI:** 10.3390/jcm15145546

**Published:** 2026-07-15

**Authors:** Giovanni Cervini, Francesca Totis, Ankita Das, Alekos Theologis, Mohammad Daher, Kyriakos D. Chatzis, Peter Tretiakov, Peter G. Passias

**Affiliations:** 1 Department of Internal Medicine, Stony Brook Southampton Hospital, Southampton, NY 11968, USA; giovanni.cervini@stonybrookmedicine.edu; 2 Division of Spine, Department of Orthopedic and Neurological Surgery, Duke University School of Medicine, Durham, NC 27705, USA; francescatotis1@gmail.com (F.T.); chatzisk10@gmail.com (K.D.C.); petertretiakov@gmail.com (P.T.); 3 Division of Spine, Department of Orthopaedic Surgery, University of California San Francisco, San Francisco, CA 94143, USA

**Keywords:** deglutition disorders, spinal fusion, cervical vertebrae, frailty

## Abstract

**Background:** Dysphagia, a common and significant complication of cervical spine surgery, remains challenging to predict. This study aims to examine baseline patient characteristics and intraoperative risk factor influence on the incidence of dysphagia following adult cervical deformity (ACD) surgery. **Methods:** Retrospective Cohort Study of a Prospectively Enrolled Database. Patients with complete baseline (BL) and 2-year (2Y) follow-up data were analyzed. Dysphagia was defined by postoperative reports or SWAL-QOL scores < 25th percentile. Descriptive statistics, means comparison tests, cross-tabulations, and regression analyses were performed. **Results:** Of 265 patients included (mean age: 58.2 ± 11.4 years, BL BMI 28.5 ± 7.6 kg/m^2^, CCI: 0.93 ± 1.3, BL frailty: 0.2 ± 0.1, operative time: 348 ± 194.9 min, and levels fused: 5.9 ± 3.6), 82 (30.9%) reported postoperative dysphagia. The dysphagia group demonstrated significantly greater BL frailty score (*p* < 0.001), BL C2–C7 (*p* = 0.002), and cSVA (*p* = 0.001). Longer operative time was associated with higher dysphagia risk (OR 1.004, *p* < 0.001). Anterior osteotomy at any level from C3–C7 demonstrated greater dysphagia rates (*p* < 0.02, all). Significant corrections of both C2–C7 and McGregor’s Slope (MGS) predict dysphagia (OR 3.6, *p* < 0.001; OR 17.0, *p* = 0.009). Delayed extubation postoperatively significantly increased occurrence (80.0% vs. 20.0%, *p* = 0.015; OR 9.5, *p* = 0.047). Dysphagia appeared to be associated with early DJF (*p* = 0.034) and worse M3 NSR scores (*p* < 0.001). A multivariable logistic regression showed operative duration to be independently associated with postoperative dysphagia (OR 1.004 per minute, 95% CI 1.002–1.005, *p* < 0.001). The surgical approach was significant overall (Wald χ^2^ = 6.44, df = 2, *p* = 0.040). However, compared with the anterior approach, neither the posterior nor the combined approach was found to be significantly associated with dysphagia (OR 0.95, 95% CI 0.40–2.28, *p* = 0.915; OR 2.11, 95% CI 0.81–5.48, *p* = 0.125). **Conclusions:** Dysphagia is associated with greater baseline frailty or deformity, undergoing mid-lower cervical osteotomies, significant correction, operative duration, or delayed extubation. Further investigation is warranted, given the small sample size of patients with delayed extubation. These findings underscore the importance of preoperative planning to mitigate dysphagia risk.

## 1. Introduction

Dysphagia is a frequent and particularly impactful complication of cervical surgical interventions, markedly affecting patients’ quality of life, not only on a nutritional level but also from a social perspective. Postoperative dysphagia can trigger a range of distressing psychosocial responses, including anxiety, shame, embarrassment, fear, and diminished self-esteem [1,2]. Patients with adult cervical deformity (ACD) are a particularly vulnerable cohort in that their condition is already known to severely impact mental health and quality of life; as such, any complication such as dysphagia that could particularly worsen their postoperative trajectory could significantly hamper the benefits expected to be conferred by surgery [3,4].

Potential causes of dysphagia following surgical interventions include traction on the laryngopharyngeal complex leading to focal pharyngeal swelling, denervation of the recurrent laryngeal or superior laryngeal nerves, and neuropraxia of the pharyngeal plexus, which may impair both motor and sensory functions of the esophagus, trachea, and nearby structures [5]. Despite its prevalence, predicting the onset and progression of dysphagia remains challenging.

Current literature reports a relatively high incidence of dysphagia following corrective surgery for cervical deformity, ranging from 11.5% to 14.3% [6,7,8]. Current research primarily addresses dysphagia in populations mainly comprising patients with degenerative cervical disease. To date, there appears to be no comprehensive analysis of risk factors associated with the development of postoperative dysphagia following adult cervical deformity corrective surgery. The paucity across deformity literature reveals a gap in our capability to effectively predict and mitigate this adverse outcome. Enhanced knowledge of the prevalence, risk factors, and associated outcomes would facilitate the development of specific preventive strategies aimed at reducing the incidence of this debilitating complication following surgery and promoting more efficient identification and allocation of supportive resources for those experiencing dysphagia.

The primary objective of this study was to examine the influence of baseline patient characteristics and intraoperative risk factors on the incidence of postoperative dysphagia following ACD surgery. Additionally, the secondary objective focuses on identifying other clinical and radiographic predictors that may influence the development of dysphagia after surgery.

## 2. Materials and Methods

### 2.1. Data Source and Study Design

The present study was conducted as a prospectively enrolled single-center database originally dating to 2012, with ongoing data collection and subsequent retrospective cohort review. Institutional Review Board (IRB) approval was obtained prior to study facilitation, and all patients provided informed consent independent of consent for the planned operation. Patients eligible for database participation must be older than 18 years and already have active plans for cervical deformity correction. Cervical deformity was defined by previously validated and frequently employed radiographic parameters that quantified the degree of coronal and sagittal malalignment. Patients, via radiograph, must demonstrate at least one of the following: C2–C7 sagittal kyphosis > 15°, T1 slope minus cervical lordosis (TS-CL) > 35°, C2–C7 SVA (cSVA) > 40 mm, chin–brow vertical angle (CBVA) > 25°, McGregor’s slope (MGS) > 20°, or segmental cervical kyphosis >15° across any 3 vertebrae between C2 and T1. All patients must also have plans to undergo correction with an upper instrumented (UIV) above the C7 vertebra. Inclusion criteria of the present study require complete radiographic and quality-of-life data preoperatively and at 2-year follow-up. Patients with any record of preoperative dysphagia were excluded from the study.

### 2.2. Cohort Designation

Patients who had a recorded diagnosis of dysphagia by another healthcare professional in the recovery phase, patients who self-reported dysphagia, or patients who met criteria for dysphagia on SWAL-QoL patient-reported outcomes measures (PROMs) constituted the dysphagia cohort. Those who registered a score < 25th percentile were considered to meet dysphagia criteria. This broader, more sensitive case definition was intentionally employed to maximize identification of patients with any degree of swallowing dysfunction, enabling a more comprehensive risk factor analysis. Furthermore, differences in follow-up duration and the clinical complexity of a deformity-specific surgical cohort may exist compared to prior studies.

### 2.3. Radiographic Assessment

At baseline and predetermined follow-up intervals (six weeks (6W), six months (6M), one year (1Y), and two years (2Y)), static and dynamic plain films with lateral and anteroposterior (AP) views, as well as full-length standing films (EOS) were obtained (EOS^®^ imaging, Paris, France). Radiographic images were subsequently analyzed using Surgimap software, v2.3.2.1 (Nemaris Inc.^TM^, New York, NY, USA). Patients were evaluated based on the morphological characteristics and severity of their cervical deformity [9,10]. Cervical sagittal alignment was comprehensively assessed by utilizing the Ames cervical modifiers and Passias redefined modifier cutoffs, using the C2–C7 Cobb angle for cervical lordosis (CL), chin–brow vertical angle (CBVA: angle between a line from the brow to the chin to the vertical when patients stands upright with both hips and knees extended), cervical SVA (cSVA: C2 plumb line offset from the posterosuperior corner of C7), and the mismatch between T1 slope and CL (TS-CL) [11,12]. Additional sagittal alignment radiographic measurements were taken to assess the patient’s cervical deformity-specific frailty score, including McGregor’s slope (MGS), C2–T3 angle, and C2 slope (C2S) [12].

### 2.4. Quality of Life Quantification

Health-related quality of life (HRQL) measures were routinely collected at baseline and follow-up visits; these tools included the aforementioned SWAL-QoL questionnaire, the Neck Disability Index (NDI), the Numerical Rating Scale for Neck (NRS-Neck), the modified Japanese Orthopedic Association score (mJOA), and the Euroqol 5D (EQ-5D). The SWAL-QoL, a scale of 0–100 (worst–best), was designed to evaluate eight dysphagia-related quality of life domains: general burden, eating duration, eating desire, food selection, communication, fear of eating, social functioning, and mental health, and two additional general quality of life domains, sleep and fatigue [13,14]. The SWAL-QoL has been previously validated amongst the cervical spine population [13,15,16]. NDI and NRS-Neck are also commonly applied and well-validated measures to capture perceived disability in patients with neck pain [17,18]. The mJOA offers the unique capacity to specifically assess the severity of myelopathy [19,20]. The cervical-specific frailty score was calculated using radiographic thresholds based on moderate and severe mJOA scores, categorizing patients into low, moderate, and severe deformity groups for each patient-reported modifier [12]. Lastly, the EQ-5D helps providers visualize the clinical impact of the condition, especially with regard to the mobility, self-care, usual activities, pain/discomfort, and anxiety/depression dimensions [4].

### 2.5. Additional Outcomes of Interest

As multi-level fusion is a known risk factor for the occurrence of distal junctional kyphosis (DJK), any instances of DJK postoperatively were documented and explored [21]. Distal junctional kyphosis (DJK) has been previously defined as the development of a kyphotic angulation greater than 10 degrees below the lowest instrumented vertebra and construct [21,22].

### 2.6. Statistical Testing

Descriptive statistics were utilized to summarize the study population and individual cohorts. Frequency distributions were calculated for pertinent demographic, surgical, radiographic, and clinical variables. Means comparison tests, such as the unpaired independent Student’s *t*-test, were utilized for nominal variables, and χ-squared cross-tabulations were applied for categorical variables. Binary logistic regression analyses helped identify any existing associations and the strength of predictors of postoperative dysphagia. Linear regression demonstrated the relationship between dysphagia and postoperative HRQL values. Given the heterogeneous cohort regarding surgical approach, a multivariable analysis was conducted. The multivariable analysis was constructed to simultaneously evaluate the contributions of surgical approach (posterior, anterior, and combined approach) and operative duration to dysphagia risk. Variables were selected a priori based on clinical relevance and univariable significance. All analyses were conducted with SPSS software (IBM Corp., IBM SPSS Statistics for Windows, v25.0. Armonk, NY, USA). For all relevant tests, two-tailed statistical significance was set to *p* < 0.05.

## 3. Results

### 3.1. Cohort Overview

Amongst the 265 patients who met the inclusion criteria, the mean age was 58.2 ± 11.4 years, the mean BMI was 28.5 ± 7.6 kg/m^2^, the mean CCI was 0.9 ± 1.3, and the mean frailty score was 0.2 ± 0.1. Of these patients, 129 (51.2%) were female. In total, 48.3% patients had some prior history of cervical intervention. Hypertension (16.9%) was the most common baseline comorbidity, followed by diabetes (14.0%). A total of 82 (30.9%) patients developed dysphagia postoperatively. There were no significant differences in baseline demographic factors, except that the dysphagia cohort demonstrated significantly greater BL frailty than the non-dysphagia cohort (0.3 vs. 0.2, *p* < 0.01). There also did not appear to be any difference between cohorts with regard to smoking history. However, the dysphagia group had a lower rate of patients with a history of hypertension (26.3% vs. 1.3%, *p* < 0.001). In terms of etiology, across the overall cohort, 82 (32.8%) patients had focal kyphosis, 8 (3.1%) patients had flat neck, and 14 (5.3%) patients had cervicothoracic origin of deformity, with the remaining not further specified. There was a significantly greater proportion of patients with focal kyphosis deformity as the primary etiology who eventually developed dysphagia as opposed to those without focal kyphosis 54.9% vs. 22.0%, *p* < 0.001) (Table 1).

### 3.2. Surgical Details

The mean operative time was 348.0 ± 194.9 min, the mean EBL was 834.5 ± 1180.3 milliliters, and the mean levels fused were 5.9 ± 3.6 levels. The mean length of stay (LOS) was 4.9 ± 5.6 days. The dysphagia cohort underwent significantly longer procedures (446 vs. 306 min, *p* < 0.001). There was no significant difference in the levels fused between the two cohorts. There was no significant difference amongst those who had previously had cervical surgery from any approach. There were greater applications of anterior osteotomies at every level from C3 to C7 amongst those who developed dysphagia (*p* < 0.01). Those with dysphagia also necessitated significantly longer lengths of stay (6.9 vs. 4.3 days, *p* = 0.005) (Table 1).

### 3.3. Postoperative Outcomes

At three months (M3), two (0.8%) patients had developed DJF, with another one (0.4%) patient demonstrating DJF by Y1. There were no significant differences for DJK development alone, but a significantly greater rate of dysphagia amongst those who experienced DJF by M3 (*p* = 0.034). There also appeared to be an increased rate of reoperation by 2 years among patients with dysphagia compared with those without dysphagia (15.9% vs. 3.3%, *p* < 0.001).

### 3.4. Impact of Degree of Correction

At baseline, the overall cohort was characterized by the following radiographic parameters: C2–C7: −7.3 ± 14.3; T1S: 29.5 ± 14.3; TS-CL: 28.2 ± 14.3; cSVA: 2.0 ± 3.2; C2S: 30.9 ± 20.2. Prior to intervention, the dysphagia group demonstrated significantly greater BL C2–C7 (−2.0° vs. −10.0°, *p* = 0.002), greater cSVA (34.0 mm vs. 11.7 mm, *p* < 0.001), and were more often considered to have severe MGS (12.2% vs. 4.9%, *p* = 0.034) (Table 1). Intraoperatively, correction of both C2–C7 and MGS parameters from severe to moderate/low categories was a significant predictor of developing dysphagia (OR 3.6, *p* < 0.001; OR 17.0, *p* = 0.009).

### 3.5. Evolution of HRQLs

At BL, the mean NSR Back was 5.8 ± 3.2, NSR Neck 7.0 ± 2.8, mJOA 13.1 ± 2.8, and EQ-5D 5.8 ± 5.0, with EQ-5D VAS of 62.4 ± 22.3. At M3, patients who had experienced dysphagia demonstrated worse NSR scores (5.9 vs. 3.7, *p* < 0.001) in addition to poorer EQ-5D VAS scores, although the difference was not significant (Table 1). The impact of HRQLs appeared to normalize between the two populations over time, with no other major differences in HRQLs at the remaining follow-up timepoints.

### 3.6. Predictors of Dysphagia

In terms of preoperative identification of at-risk patients, greater frailty at BL drastically increased the likelihood of developing dysphagia postoperatively (OR 741.8, CI 95% 69.6–7912.2, *p* = 0.001). Those with morphologic focal kyphosis at BL were more likely to experience dysphagia (OR 4.3, CI 95% 2.4–7.6, *p* < 0.001). Increasing operative time was also associated with a higher likelihood of acquiring dysphagia (OR 1.004, CI 95% 1.002–1.006, *p* < 0.001). Those who underwent a posterior-only approach had a decreased likelihood of developing dysphagia (OR 0.56, CI 95% 0.33–0.95, *p* = 0.03), while those who underwent a combined anterior–posterior approach demonstrated increased risk of dysphagia (OR 2.64, CI 95% 1.49–4.67, *p* < 0.001). Those who had an osteotomy from an anterior approach at any level from C3–C7 demonstrated significantly greater rates of dysphagia (*p* < 0.02, all). Loss of cervical lordosis also increased the likelihood of dysphagia (OR 3.6, CI 95% 1.7–7.5, *p* < 0.001). Perioperatively, patients who could not be successfully extubated immediately postoperatively demonstrated significantly greater occurrence of dysphagia (80.0% vs. 20.0%, *p* = 0.015); failure to extubate increased the odds of dysphagia (OR 9.5, *p* = 0.047). Of note, these results warrant cautious interpretation given the small sample size of delayed extubation. See Table 2 for further associations. A multivariable logistic regression showed operative duration to be independently associated with postoperative dysphagia (OR 1.004 per minute, 95% CI 1.002–1.005, *p* < 0.001). The surgical approach was significant overall (Wald χ^2^ = 6.44, df = 2, *p* = 0.040). However, compared with the anterior approach, neither the posterior nor the combined approach was found to be significantly associated with dysphagia (OR 0.95, 95% CI 0.40–2.28, *p* = 0.915; OR 2.11, 95% CI 0.81–5.48, *p* = 0.125).

## 4. Discussion

Dysphagia is a distressing complication following cervical deformity corrective surgery, potentially disrupting daily and social life, reducing satisfaction with the surgery, and diminishing quality of life if it persists. In this study, we aimed to identify patient-specific, perioperative, and postoperative risk factors associated with the development of postoperative dysphagia in patients who underwent cervical deformity corrective surgery.

Amongst preexisting patient-specific factors, we found that patients with greater frailty at baseline were more likely to develop postoperative dysphagia. Given large confidence intervals of the OR, interpretation of these results should be conducted cautiously. Additionally, specific preoperative deformity characteristics and radiographic parameters were strongly correlated with the development of postoperative dysphagia. For example, patients with morphologic focal kyphosis at baseline were more likely to experience dysphagia (OR 4.3, *p* < 0.001). This increased risk is likely due to an acute bend in the mid-esophagus, which predisposes individuals to difficulties with swallowing. Notably, patients with a greater baseline C2–C7 angle (*p* = 0.002) and cSVA (*p* = 0.001) were found to be more likely to develop postoperative dysphagia. As such, we hypothesize that cervical deformity corrective surgery could lead to considerable temporary or even long-term changes in the anatomical relationship between the cervical spine and the anterior esophagus, potentially inducing dysphagia.

Additionally, our findings indicate that the extent of surgical correction significantly influences the likelihood of dysphagia. Specifically, corrections that alter the C2–C7 and MGS from severe to moderate or low were significant predictors of dysphagia. Interestingly, these radiographic parameters were also identified as risk factors for postoperative dysphagia in other types of cervical spinal surgery [23,24,25,26]. Hypertension was not associated with dysphagia postoperatively (non-dysphagia group 26.3% vs. dysphagia group 1.3%, *p* < 0.001). This inverse association may suggest antihypertensive medications as a potential confounding variable, with possible protective effects in this patient population; however, this could not be evaluated in the present study. Alternatively, this finding may reflect sampling variation or a type I error. Further investigation is warranted to clarify this relationship. Overall, these patient-specific factors can be used to prognosticate postoperative dysphagia during preoperative counseling and to facilitate perioperative resource allocation.

Amongst operative factors, our findings indicate that increased operative time is associated with a higher likelihood of postoperative dysphagia. Perioperatively, time to extubation seemed to be linked to the development of dysphagia, with failure to extubate increasing the odds of dysphagia by 9.5 times. This finding should be interpreted cautiously, given the small number of patients with delayed extubation. As such, this observation is at best hypothesis-generating and may suggest that prolonged intubation could increase the risk of direct or indirect injury to the nerves, trachea, and pharyngeal muscle complex during surgery. Further investigation is warranted to clarify this relationship. These observations suggest that prolonged intubation may elevate the risk of direct and indirect injuries to the nerves, trachea, and pharyngeal muscle complex during surgery. Such injuries can lead to oropharyngeal muscle inactivity, glottic injury, mucosal inflammation resulting in the loss of tissue architecture, and vocal cord ulcerations, all of which contribute to swallowing dysfunction. Another significant operative factor is the performance of an osteotomy via an anterior approach at any level from C3 to C7, which was associated with significantly higher rates of dysphagia. This could be related to the anterior approach itself or the degree of angular pressure that then distributes pressure differently across key anterior neck compartment structures. This is supported by the observation that although the anterior approach was not isolated as a risk factor itself, the risk appears to significantly increase when comparing posterior-only approaches to combined anterior–posterior ones. We hypothesize that the dissection of soft tissue during exposure of the anterior column may lead to increased damage to the esophagus and nerves, thereby resulting in higher dysphagia rates due to disruptions in swallowing mechanics. Specifically, surgeries performed at the C3 level pose risks to the glossopharyngeal and hypoglossal nerves, which are crucial for the oral phase of swallowing [27]. Additionally, the superior laryngeal nerve and the recurrent laryngeal nerve, which are vital for the pharyngeal phase, can be exposed during surgeries involving the C3 and C4 levels [28]. The esophageal phase may also be compromised by postoperative neck swelling, prominent hardware such as anterior plates, and possible esophageal ischemia [29]. Furthermore, the degree of correction of certain radiographic parameters appeared to influence the likelihood of dysphagia, perhaps reflecting the natural tendency to compensate for physiological alignment around the present alignment. Deviations from such alignment may then require recalibration of certain physiological functions such as swallowing.

While operative time is suggested as a risk factor, what was not immediately clear was whether this was due to confounding factors associated with the surgical approach. A multivariable analysis was performed to account for this possibility, given that certain approaches may be associated with greater procedural complexity and duration. Evaluating these variables simultaneously allowed assessment of their associations with postoperative dysphagia. Our analysis showed operative duration remained associated with postoperative dysphagia. However, the surgical approach, while overall statistically significant, did not demonstrate consistent differences between groups. This suggests that operative time, whether due to procedural complexity or exposure time, may explain differences observed across surgical techniques in a more meaningful way than approach selection alone.

Among postoperative factors, we identified a significant correlation between the occurrence of dysphagia and the development of DJF by M3 post-surgery (*p* = 0.034). Furthermore, in terms of HRQL, the cohort experiencing dysphagia reported a significantly worse Neck Disability Index score at M3 (*p* < 0.001). While DJF was statistically significant, the small sample size warrants cautious interpretation of these findings. Nevertheless, the significance of DJF should not be overlooked, and a more detailed study is necessary. Further investigation may suggest that dysphagia not only significantly impacts the quality of life in the immediate postoperative period but also has the potential to alert providers to the likelihood of debilitating radiographic complications. Perhaps greater therapy resources and more frequent follow-up and surveillance in this short-term period could minimize the strain in this vulnerable period.

To date, no studies have specifically evaluated the potential use of intraoperative steroids for mitigating the risk of dysphagia following cervical deformity corrective surgery. However, insights can be drawn from methods employed in other types of cervical spine surgery. One such approach is the use of intraoperative steroids, which have been associated with a lower incidence of dysphagia in the early to intermediate postoperative period, according to most data [30]. For instance, a prospective single-blind randomized controlled trial involving 75 patients compared outcomes among those receiving local steroid administration, intravenous (IV) steroid administration, and no steroid administration intraoperatively [31]. At 2 weeks postoperatively, the local steroid group exhibited a significantly lower prevalence of severe dysphagia, based on EAT-10 scores, compared with the control and IV steroid groups. However, both steroid groups showed significantly less severe dysphagia compared with the control group at 6-week and 3-month follow-ups. At 1-year postoperatively, both steroid groups continued to show significantly reduced dysphagia rates compared to the control group. Another potential strategy involves esophageal manipulation prior to surgery. A prospective randomized controlled trial of 102 patients assessed the effects of tracheoesophageal traction exercises conducted for three days preoperatively [32]. The patients undergoing 2- to 4-level anterior cervical surgical procedures and participating in traction exercises demonstrated better Bazaz dysphagia scores in the first three weeks postoperatively compared to the control group. However, by 6 weeks and at later follow-up evaluations, there were no differences in dysphagia rates between the groups. These methods, along with other strategies such as the thoughtful selection of implant design, choice of surgical approach, and the application of multimodal methods, could potentially decrease the rate of postoperative dysphagia. This realm may also entail opportunities for multidisciplinary collaboration with otolaryngology surgeons who, at some institutions, anecdotally provide support such as pressure checks during cases involving anterior approaches. Further research in these areas is highly desirable and may reduce the incidence of postoperative dysphagia and improve outcomes for patients undergoing cervical deformity surgery.

This study is subject to several limitations. Firstly, the assessment of postoperative dysphagia relied primarily on patient-reported outcomes, without objective verification using tools such as video fluoroscopic swallowing tests or laryngoscopic assessments of vocal cord function, as these were not part of the standard clinical protocol for this cohort. In addition, the heterogeneity of a composite definition of dysphagia carries the risk of outcome misclassification and potential incidence overestimation. Secondly, this study did not perform a formal temporal analysis of dysphagia trajectory, including time to resolution, proportion with persistent symptoms, and recovery curves. Future prospective studies should incorporate standardized reassessment protocols and time-to-resolution analyses to better characterize the natural history of dysphagia following surgical intervention. The retrospective nature of the analysis limits the strength of the evidence compared to prospective studies. As a result, our conclusions require validation through future randomized controlled trials. It is also apparent that this study had a small sample of patients who underwent delayed extubation. The statistical instability of the delayed extubation OR requires further investigation utilizing a larger sample size to completely understand its effects. Sensitivity analyses stratifying dysphagia cases by capture method were not feasible given case overlap and sample size constraints; thus, they should be conducted in future studies. The observed odds ratio for frailty should be interpreted with caution, given evidence of quasi-complete separation, suggesting instability within the model. Future studies with larger sample sizes are needed to confirm this association and may benefit from penalized regression approaches to mitigate separation-related bias. In this study, the multivariable analysis was limited to surgical approach and operative duration, and did not account for additional potential confounding variables. This intentional focus, aimed at improving statistical power in a heterogeneous cohort, represents a limitation of the analysis. The observational design of this study precludes the establishment of causal relationships. To confirm our findings and enhance their generalizability, further studies, ideally multi-center and randomized controlled trials, are necessary. Lastly, the etiology of the cervical deformity was not recorded.

## 5. Conclusions

Dysphagia may occur in patients who exhibit greater frailty or deformity at baseline, those who undergo osteotomies from the mid-lower cervical region, and those who receive a significant degree of correction from baseline. Furthermore, delays in extubation may considerably increase the risk of dysphagia, but further study is necessary. Of note, these results warrant cautious interpretation given the small sample size of delayed extubation, and further investigation is necessary to completely understand the effects of delayed extubation using a larger sample size. Ultimately, dysphagia demonstrates alarming comorbidity with radiographic failure and poor short-term clinical outcomes. Given this association, preoperative risk stratification using frailty scoring and radiographic deformity assessment, multidisciplinary preoperative counseling for high-risk patients, consideration of speech–language pathology consultation in patients with delayed extubation or significant correction, and heightened postoperative surveillance for dysphagia symptoms, particularly in the early postoperative period, are clinically significant strategies to consider for reducing the risk of dysphagia.

## Figures and Tables

**Table 1:** Perioperative differences between patients with and without dysphagia.

Variable	No Dysphagia (*n* = 183)	Dysphagia (*n* = 82)	*p*-Value
	**Baseline [BL] Preoperative/Preexisting**		
Age (years)	58.4 ± 11.8	57.6 ± 10.6	0.60
Gender (Females)	83 (48.8%)	46 (56.1%)	0.28
Charlson Comorbidity Index [CCI]	1.0 ± 1.3	0.9 ± 1.4	0.54
BL frailty	0.2 ± 0.1	0.3 ± 0.2	<0.001
Prior cervical intervention	38 (47.5%)	34 (49.3%)	0.83
Focal Kyphosis	37 (22.0%)	45 (54.9%)	<0.001
Flatneck	6 (3.4%)	2 (2.4%)	0.677
Cervicothoracic	9 (5.1%)	5 (6.1%)	0.754
Smoking	10 (20.0%)	8 (23.5%)	0.70
Hypertension	35 (26.3%)	1 (1.3%)	<0.001
Diabetes	21 (12.5%)	14 (17.1%)	0.33
	**Intraoperative**		
Operative time (minutes)	307 ± 170	447 ± 218	<0.001
Anterior approach	28 (16.7%)	11 (13.4%)	0.51
Posterior approach	98 (58%)	36 (44%)	0.033
Combined	37 (22%)	35 (42.7%)	0.001
Levels fused	5.8 ± 3.7	6.1 ± 3.4	0.533
EBL	775 ± 1274	967 ± 937	0.31
LOS	4.3 ± 3.7	6.9 ± 9.2	0.005
	**Correction-related**		
Severe BL McGregor Slope [MGS]	9 (4.9%)	10 (12.2%)	0.03
BL C2–C7 lordosis	−10.0 ± 17.8	−2.0 ± 18.4	0.002
BL cSVA	11.7 ± 34.8	34.0 ± 20.0	<0.001
	**Postoperative**		
Delayed extubation	2 (1.1%)	4 (4.9%)	0.06
BL NSR neck	6.6 ± 3.0	7.5 ± 2.2	0.02
BL Months EQ-5D VAS	61.9 ± 21.6	63.1 ± 23.5	0.72
3 Months NSR neck	3.7 ± 3.4	5.9 ± 2.8	<0.001
3 Months EQ-5D VAS	67.8 ± 22.5	59.7 ± 21.8	0.06

**Table 2:** Strengths of Association with Dysphagia.

Factor	Odds Ratio [OR] (Confidence Interval [CI] 95%)	*p*-Value
**Baseline [BL] Preoperative/Preexisting**		
Age (years)	0.99 (0.97–1.01)	0.602
Gender	1.34 (0.79–2.23)	0.280
Charlson Comorbidity Index [CCI]	0.92 (0.70–1.20)	0.54
**BL frailty**	**741.8 (69.6–7912.2)**	**0.001**
**Focal Kyphosis**	**4.31 (2.44–7.60)**	**<0.001**
Flatneck	0.71 (0.14–3.59)	0.677
Cervicothoracic	1.20 (0.39–3.69)	0.754
**Intraoperative**		
**Operative time (minutes)**	**1.004 (1.002–1.006)**	**<0.001**
Anterior approach	0.78 (0.37–1.65)	0.507
**Posterior approach**	**0.56 (0.33–0.95)**	**0.033**
**Combined**	**2.64 (1.49–4.67)**	**<0.001**
Levels fused	1.02 (0.95–1.10)	0.533
**Correction-related**		
Severe BL McGregor Slope [MGS] *	**2.69 (1.04–6.88)**	**0.04**
**Δ MGS**	**17.0 (2.05–140.46)**	**0.009**
**Δ C2–C7 lordosis**	**3.61 (1.74–7.50)**	**<0.001**
Δ cSVA	1.09 (0.90–1.31)	0.397
**Postoperative**		
**Delayed extubation**	**9.33 (1.03–84.9)**	**0.047**

Note. * Less than −12° or greater than 19° is considered to be severe McGregor Slope (MGS) per Passias et al. 2021 [12]. Loss of lordosis is also defined as a transition from severe (<21°) to low (>3°) categories per the above reference. The BL frailty OR was associated with a large confidence interval; therefore, cautious interpretation is warranted, given evidence of quasi-complete separation and resulting model instability.

## Data Availability

The raw data supporting the conclusions of this article will be made available by the authors on request.
